# Three-Dimensional Scaffold from Decellularized Human Gingiva for Cell Cultures: Glycoconjugates and Cell Behavior

**Published:** 2013-07-02

**Authors:** Somayeh Naderi, Jina Khayat Zadeh, Nasser Mahdavi Shahri, Khadijeh Nejad Shahrokh Abady, Mojtaba Cheravi, Javad Baharara, Seyed Ali Banihashem Rad, Ahmad Reza Bahrami

**Affiliations:** 1 Department of Biology, Faculty of Science, Mashhad Branch, Islamic Azad University, Mashhad, Iran; 2 Department of Periodontics, School of Dentistry and Dental Research Center, Mashhad University of Medical Sciences, Mashhad, Iran; 3 Cell and Molecular Biotechnology Research Group, Institute of Biotechnology, Ferdowsi University of Mashhad, Mashhad, Iran

**Keywords:** Blastema Tissue, scaffold, Gingiva, Decellularization, Extracellular Matrix

## Abstract

**Objective:**

We studied both the presence of some carbohydrate compounds in a threedimensional (3D) matrix harvested from human gingiva and the cell behavior in this matrix.

**Materials and Methods:**

In this experimental research, in order to prepare 3D scaffolds, human palatal gingival biopsies were harvested and physically decellularized by freezethawing and sodium dodecyl sulfate (SDS). The scaffolds were placed within the rings of blastema tissues obtained from a pinna rabbit, *in vitro*. We evaluated the presence of glycoconjugatesand cellular behavior according to histological, histochemical and spectrophotometry techniques at one, two and three weeks after culture. One-way analysis of variance (ANOVA)comparedthe groups.

**Results:**

Extracellular matrix (ECM) remained after decellularization of tissue with 1% SDS. Glycoconjugate contents decreased meaningfully at a higher SDS concentration (p<0.0001). After culture of the ECM scaffold with blastema, we observed increased staining of alcian blue, periodic acid-Schiff (PAS) and toluidine blue in the scaffold and a number of other migrant cells which was caused by cell penetrationinto the scaffold. Spectrophotometry results showed an increase in glycosaminoglycans (GAGs) of the decellularized scaffolds at three weeks after culture.

**Conclusion:**

The present study has shown that a scaffold generated from palatal gingival tissue ECM is a suitable substrate for blastema cell migration and activity.This scaffold maypotentially be useful as a biological scaffold in tissue engineering applications.

## Introduction

Tissue regeneration requires three components popularly referred to as the "tissue engineering triad": the scaffold, cells and growth factor. The scaffold acts as template for cell organization and tissue development in the tissue engineering process (1).

Scaffolds can be classified into two categories according to their sources,natural (e.g., collagen) and synthetic (e.g., polyglycolide) biomaterials. Synthetic biomaterials have better physical and mechanical control however because of difficulties in attachment and growth, biocompatibility is an issue. The advantages of natural scaffolds are their low immunogenicity and capacity for interaction with the host tissue (2, 3).

To create natural scaffolds from biological tissue or organs, decellularization must be performed. Efficiency of decellularization is dependent both upon the tissue’s origin and the specific chemical or physical methods used (4). Cell extraction techniques such as sodium dodecyl sulfate (SDS), Triton X-100, deoxycholic acid (DEOX) and trypsin digestions have been employed to generate a potential a cellular matrix (5).

In natural scaffolds that have been derived from decellularized tissues or organs, the allogenic and xenogeneic cellular antigens are omitted however numerous structural and functional proteins of the Extracellular matrix (ECM) are maintained. These scaffolds are successfully used in tissue engineering (6).

The use of decellularized ECM as a scaffold has been expanded to many organs, such as the heart, lungs, liver, kidneys, pancreas and intestines (7).

ECM is a molecular complex composed of collagen, elastin, glycoproteins, proteoglycans, glycosaminoglycans (GAGs) and proteins such as growth factors, cytokines, enzymes and their inhibitors (8). It has a role in the various processes of cell adhesion, growth, migration and differentiation (9).

ECM is often used for production of biological scaffolds in tissue engineering (10). The dominant elements in gingival connective tissue are fibroblasts that produce collagen, reticular and elastin fibers, glycoproteins (mainly fibronectins) and GAGs [hyaluronic acid (HA) and chondroitin sulfate] (11).

ECM molecules (especially GAGs) influence cellular behavior based on a dynamic reciprocal relationship model, interact with cell surface receptors, and after signal transduction they cause a cascade of events that lead to special gene expression whose products affect ECM in various ways (12).

Cell-cell and cell-matrix interactions are essential for biomaterial design in tissue engineering and can assist in treatment of diseases (13, 14). Therefore, with regards to the effective role of these molecules in tissue engineering, it is necessary to perform additional studies on the morphogenic signals derived from the ECM.

Recent progress has provided new opportunities for rehabilitative medicine and tissue restoration. Among the available clinical options, embryonic stem cells (ESC) derived from rabbit blastocyst inner cell mass have been studied for several years (15).

When stem cells are seeded on natural ECM scaffolds, they are instructed to differentiate into specific cell types dependent upon the source tissue of the ECM and arrange themselves into appropriate functional units (16).

Blastema tissue is a group of undifferentiated cells in some parts of healing tissue that has the capability to divide, differentiate and participate in the process of healing damaged tissues. The rabbit ear is a good model for blastema tissue studies (17).

Due to the numerous similarities between blastema cells and stem cells, therefore blastema tissue derived from a rabbit’s ear is a good model for studying cell behavior innatural scaffolds.

Additionally, numerous studies have focused on the mechanisms of cell adhesion and migration in two-dimensional models (18). However the use of three-dimensional (3D) matrices might be more suitable as a model for cell behavior studies.

The aims of the present study were initially to provide a 3D matrix (natural scaffold) from decellularized gingival tissues, followed by *in vitro* histochemical investigation of the interactions between blastema tissue and this scaffold.

## Materials and Methods

### Decellularization of gingiva tissue to produce a natural scaffold

In this experimental study, human palatal gingiva tissues were procured from patients who underwent dental treatments, restorative-prostheses and third molar surgeries at a specialized dental clinic. Tissue was acquired with the aid of a dental specialist and by taking into consideration ethical regulations. Samples were transferred to the lab in physiological serum, cut into equal pieces (5×5 mm), placed in 2-ml cryotubes and soaked for two minutes in liquid nitrogen for immediate freezing. For quick thawing, samples were placed for five minutes in distilled water; these steps were repeated six times. The samples were then placed in phosphate-buffered saline (PBS) for one hour. Next, they were divided into three groups and washed in 0.1, 0.5 and 1% SDS (CinnaGen, Iran) detergent for 24 hours. Samples were repeatedly washed with distilled water (19). Bouin’s solution was used to fix the samples. Paraffinized sections of each group were stained by hematoxylin-eosin to evaluate the success of the decellularization process. For statistical analysis, Randomly, ten slides from each sample and ten microscopic field (×40 magnification) from each slides were considered according to stereological method (20).

### Preparation of blastema tissue

For this study, 6-8 month old male white New Zealand rabbits (n=6)that weighed approximately 2.5 kg were obtained from Razi Vaccine and Serum Research Institute (RVSRI).

Animal experiments were performed according to the Iranian Council for the Use and Care of Animals Guidelines and the study was approved by the Animal Research Ethical Committee of Tehran University of Medical Sciences. Animals consumed a base food regimen and were keptin individual cages in an animal house at a controlled temperature (20 ± 2˚C) and 12 hours lighting. After anesthetizing animals with lidocaine, we created several 2 mm diameter holes in each rabbit’s pinna using a punch instrument. After 3 days, another 4 mm diameter hole was inflicted in the healing wounds and blastema rings were separated (21).

### Tissue culture

**Fig 1:**
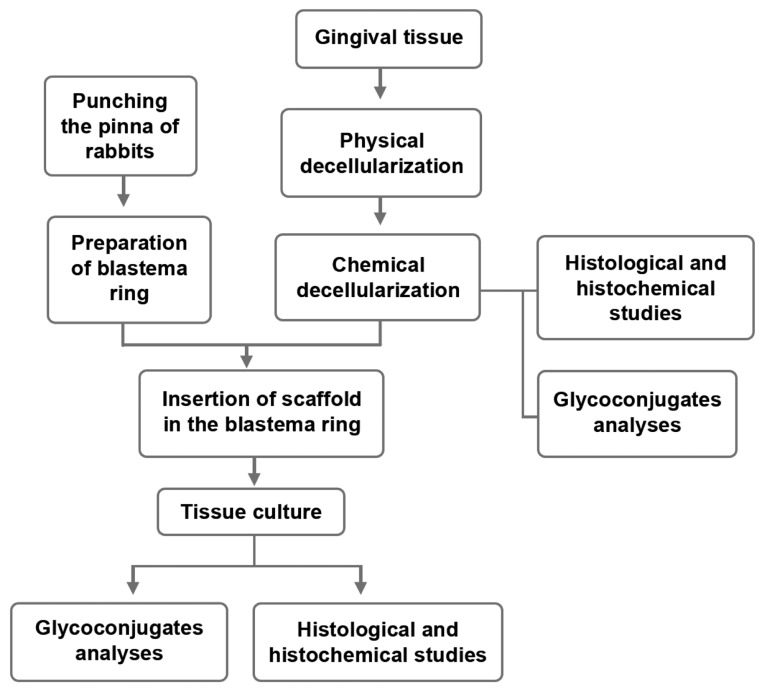
Schematic representation of the study procedures.

Scaffolds prepared from decellularized gingiva tissue were sterilized with 70% ethanol before they were inserted in the blastema ring in a laminar flow hood under asepticconditions (Fig 1). The scaffolds with the blastema ring were transferred to a 12 well-plate (Orange Scientific, Belgium) in Dulbecco’s modified Eagle,s medium (DMEM, Gibco, New York) supplemented with 15% fetal bovine serum (FBS, Gibco, Netherlands) and 100 μl penicillin/streptomycin (Biosera) and incubated for three weeks at 37˚C in 5% CO_2_ in air. For the control, scaffolds without blastema were cultured.

### Histological and histochemical studies

Cultured tissues were fixed in Bouin’s solution, dehydrated by a graded ethanol series, embedded in paraffin, and sliced into 7 μm sections by a microtome. Hematoxylin-eosin staining was performed to observe the behavior of migrant blastema cells into the scaffold. We used periodic acid-Schiff (PAS), alcian blue (8G-X, pH=2.5) and toluidine blue staining for histochemical assessment of acidic (carboxylated and sulfated) and neutral carbohydrate compounds. Color intensity (Table 1) was graded (0-5) according to the Gong et al method (22).

**Table 1:** Grading staining color intensity guideline for histochemical staining methods (22)


Color intensity	Number of exon

-	No color
+	Very weak color
++	Weak color
+++	Average color
++++	Intense color
+++++	Very intense color


For PAS, cells were allowed to oxidize for 10 minutes in periodic acid, and then washed in running water for 5 minutes, followed by immersion in Schiff’s reagent for 10 minutes. Periodic acid is used for oxidation of some of the tissue carbohydrates which produces aldehyde groups that can then condense with Schiff’s reagent to form a bright red color. This indicates the tissue component to which the neutral carbohydrate is attached.

### Glycoconjugate analyses

We determined the scaffold GAG content by the 1, 9-dimethylmethylene blue (DMMB) assay. Matrix analyses were performed before and after culture with blastema. A 100 μl of the proteinase K digested sample was mixed with 1 ml DMMB dye after which optical absorption was measured by a spectrophotometer (Scanning Spectrophotometer, UVD-2950) at 525 nm (23, 24). The results were obtained by extrapolation from a standard curve using shark chondroitin-6-sulfate.

### Statistical analysis

Data were obtained at least in triplicate (n=6), averaged and expressed as mean ± standard deviation (SD). Statistical analysis was carried out using one-way analysis of variance (ANOVA). A value of p≤0.05 was considered statistically significant.

## Results

Liquid nitrogen followed by 1% SDS for 24 hours was chosen as the best decellularization procedure for preparing a scaffold from human gingiva.

**Fig 2:**
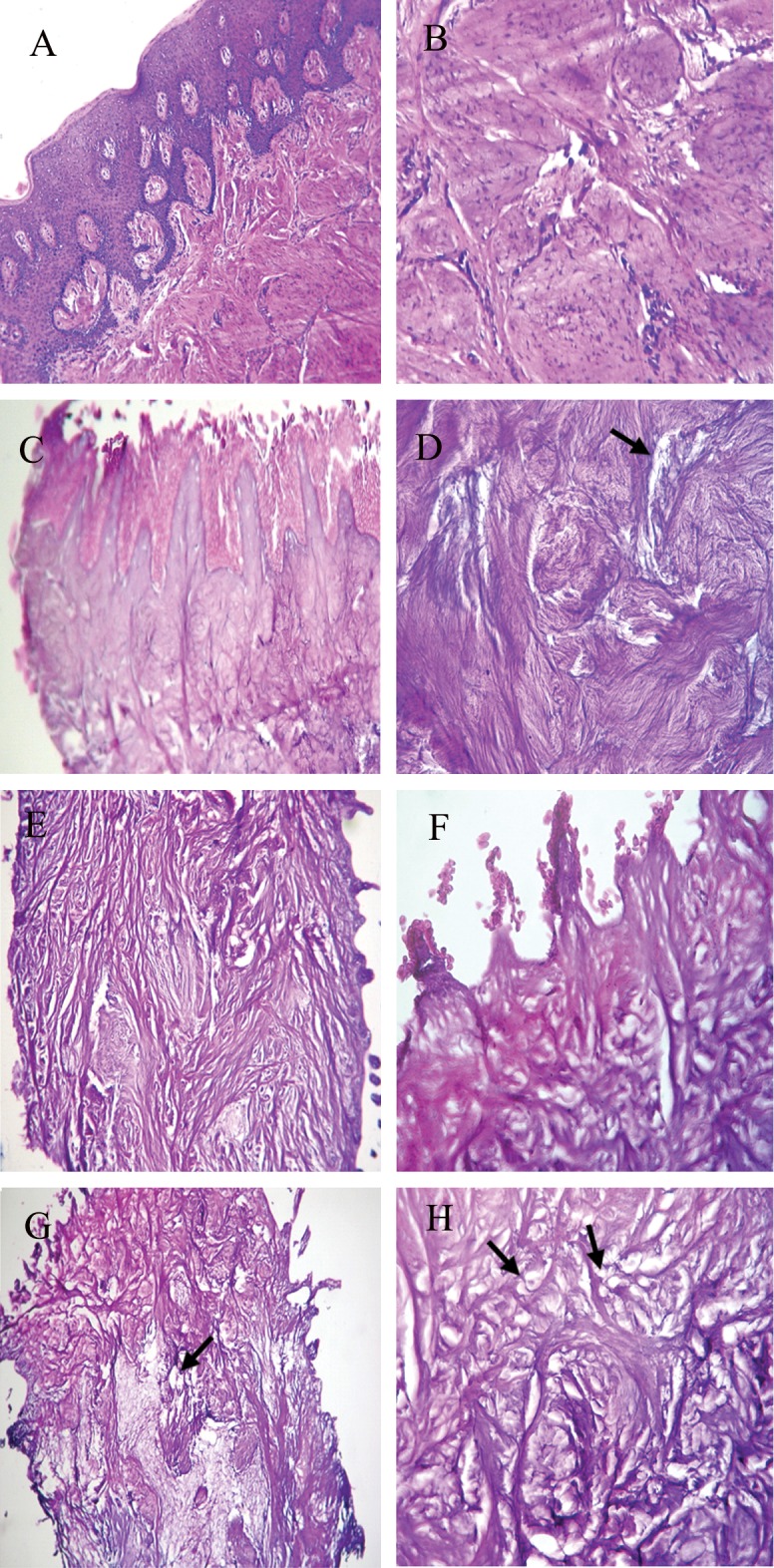
Hematoxylin-eosin stained decellularized human gingiva tissue. Gingiva tissue(A, B); decellularized human gingiva tissue with SDS 0.1% (C, D); SDS 0.5% (E, F); and SDS 1% (G, H). Magnification: ×100 (A, E, G); ×400 (B, C, D, F, H). At SDS concentration of 1%, an increase in scaffold porosity was observed (arrows).

**Fig 3:**
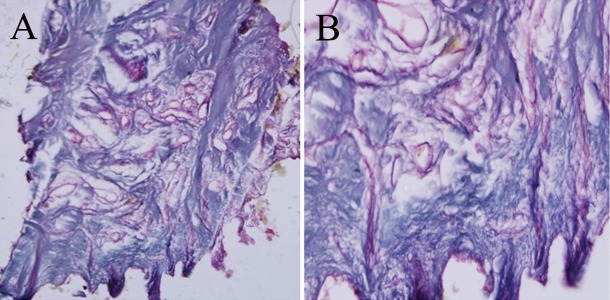
Decellularized human gingiva tissue with 1% SDS (PAS staining). A) Magnification: ×100 (A) ×400 (B).

**Fig 4:**
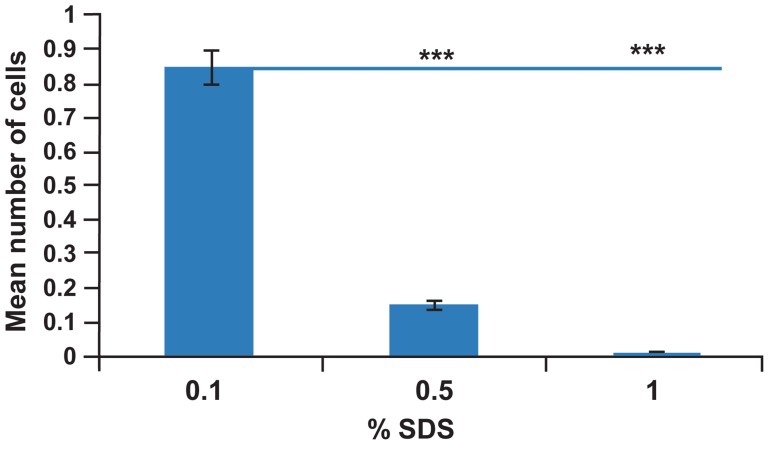
Mean number of cells in SDS decellularized gingiva matrix. By increasing SDS concentration to 1%, the decellularization rate of human gingiva tissue was significant increased. Data: Mean ± standard deviation (n=6, *** p=0.001).

**Fig 5:**
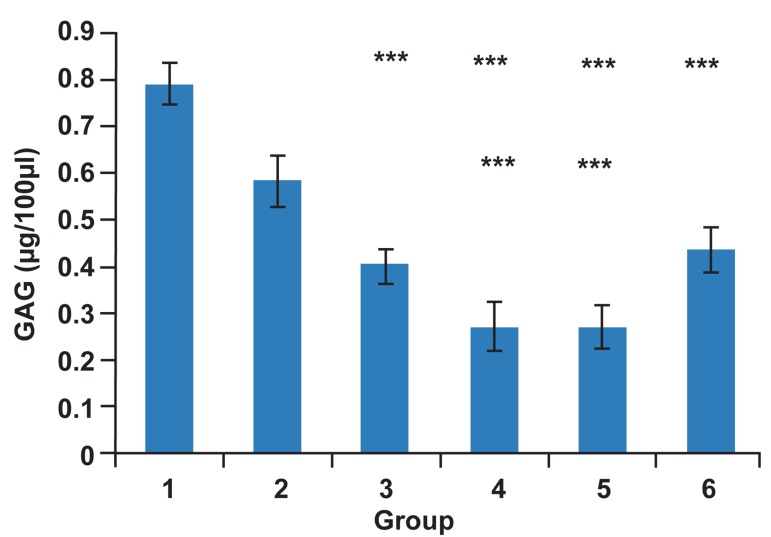
GAG content in SDS decellularized gingiva matrix and matrix after culture. 1. Gingiva tissue before decellularization. 2. 0.1% SDS. 3. 0.5% SDS. 4. 1% SDS. 5.Without blastema matrix at 20 days after culture. 6. Matrix placed in blastema ring 3 weeks after culture. GAG content Significantly decreased with increased SDS concentration. GAG content increase shown in samples after 3 weeks of culture. Data: Mean ± standard deviation (n=6, ***p<0.0001).

The results showed that after decellularization treatment with 1% SDS, the epithelial layer and connective tissue layer were removed, which resulted in increased scaffold porosity (Fig 2, 3). By increasing the SDS concentration to 1%, the decellularization rate of human gingival tissue increased (Fig 4). The response to PAS was average; however alcian blue and toluidine blue showed weak reactions. A reduction in GAG content after an increase in the SDS concentration was observed by spectrophotometry (p<0.0001). The mean ± SD, GAG content was as follows: before decellularization (0.79 ± 0.045 μg/100 μl), 0.1% SDS group (0.58 ± 0.054 μg/100 μl), 0.5% SDS group (0.4 ± 0.04 μg/100μl) and 1% SDS group (0.27 ± 0.05 μg/100 μl), (Fig 5). From ten days after blastema cells were cultured with the scaffold, we observed some penetrating blastema cells in the scaffold. We used PAS staining to evaluate the amount of polysaccharides present in the migrating cells and substrate. Blastema cells, before migration (week one after culture), showed an average response to PAS.

**Fig 6:**
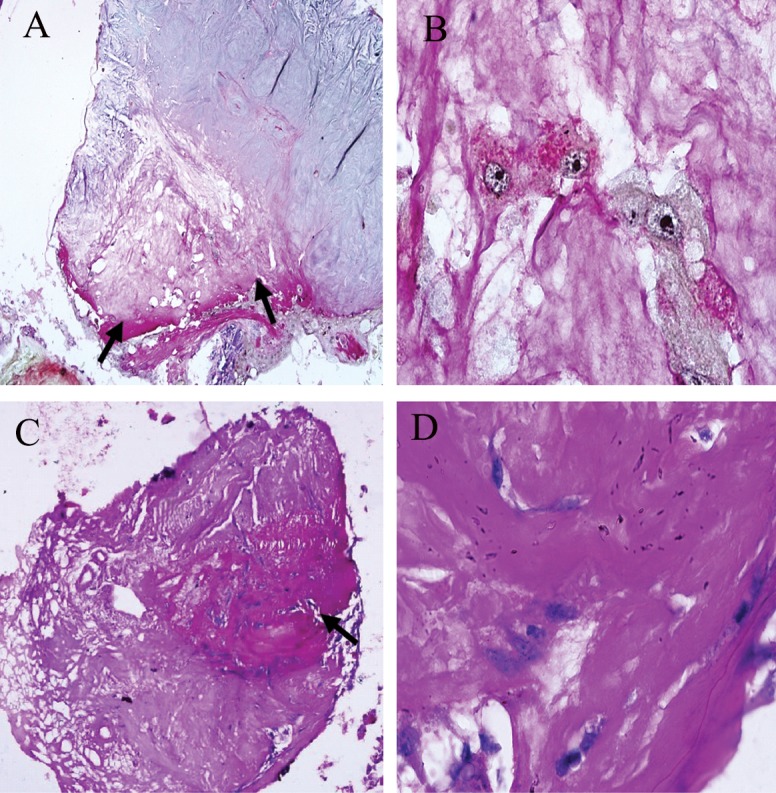
PAS staining of transverse section from blastema ring with scaffold, different weeks after culture. A.Color changes in scaffold margins due to penetration of cells at week 2 after culture asshown with arrows (magnification: ×100). B. Penetration of cells into the scaffold (magnification: ×1000). C. Transverse section from scaffold at 3 weeks after culture showing progression of response to PAS dye (Color intensity) to the scaffold’s center (magnification: ×100). D. Cells that penetrated into the scaffold (magnification: ×1000).

Howeverthe responses were very strong after penetration of the cells into the scaffold at weeks two and three after culture. The reaction of the scaffold to PAS at week one was average, but increased until the end of the culture period (Table 2). Zones of blastema cells that penetrated into the scaffold, showed strongly positive response to PAS dye (Fig 6).

**Fig 7:**
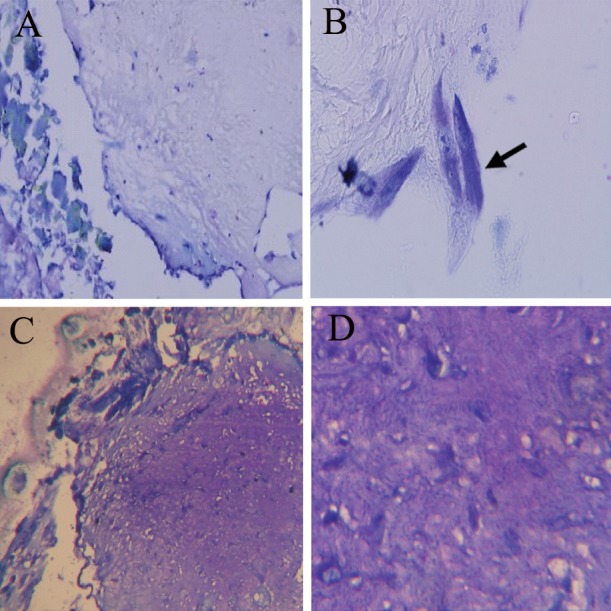
Toluidine blue stain of transverse section from the blastema ring with scaffold at weeks 2 and 3 after culture. A,B. Cells penetrated into the scaffold with very weak metachromasia (+) at 2 weeks after cultureas shown with an arrow (magnification: ×100, ×1000). C, D. Scaffold with average metachromasia response (+++), 3 weeks after culture with blastema (magnification: ×400, ×1000).

We used toluidine blue stain to determine the presence of mucopolysaccharides. The cells showed a very weak response to toluidine blue during all of the studied time periods, however the scaffold response to this dye was negative prior to cell penetration. The scaffold response was average after cell penetration at week three following culture (Fig 7).

**Fig 8:**
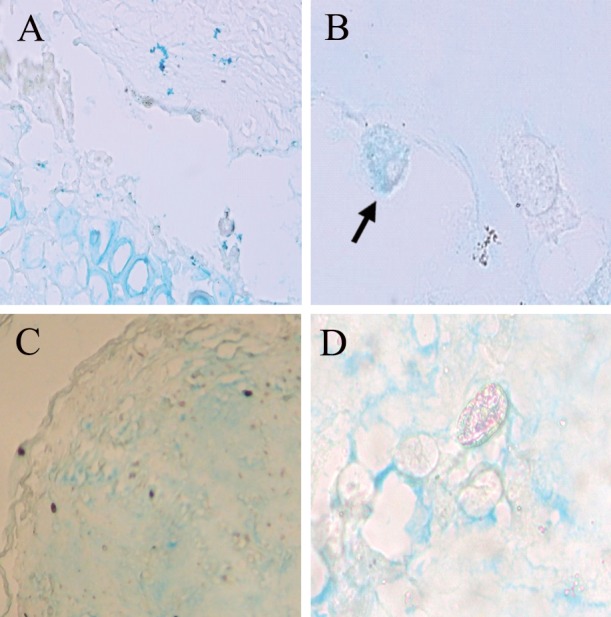
Alcian blue stain of transverse section from blastema ring with scaffold at weeks 2 and 3 after culture. A, B. 2 weeks after culture. Cell with average response (+++) is shown With arrow. C, D. Average response in some regions of the scaffold at 3 weeks after culture (magnification: ×200, ×1000).

Staining of scaffold and cells with alcian blue to detect the presence of acid mucopolysaccharides was weakly responsive in the first days of the culture. However, gradually, at two weeks after culture of the scaffold, an average response was noted.The scaffold was less responsive compared to the cells. At week three after culture, the scaffold response was average but the cell response was negative (Fig 8).Histochemical study results are shown in table 2.

Spectrophotometric studies indicated that GAGs content in the matrixes at week three after culture with blastema (0.44 ± 0.05) increased compared to the control scaffold (0.27 ± 0.05; Fig 5).

**Table 2:** Case report from staining color intensity in response to histochemical staining methods during scaffold study period


staining Culture days	PAS		Toluidine blue		Alcian blue (pH=2.5)	
	Scaffold	Cells	Scaffold	Cells	Scaffold	Cells

**Scaffold before culture**	+++	*	-	*	+	*
**Conrtol scaffold (with out blastema) after culture**	+++	*	-	*	+	*
**Scaffold 1 week after culture**	+++	+++	-	+	+	++
**Scaffold 2 week after culture**	+++++	+++++	+	+	+	+++
**Scaffold 3 week after culture**	+++++	+++++	+++	+	+++	-


*; No cells in scaffold.

## Discussion

Biologic scaffolds prepared from the ECM of decellularized mammalian tissues have been shown to facilitate constructive remodeling in injured tissues such as skeletal muscle, the esophagus and lower urinary tract, among others (25).

In decellularization experiments, maintaining an ECM and basement membrane structure and the resistance of prepared scaffold against the graft are very important (26). ECM and basement membrane structure are significant components of cellular strength and adherence. The basement membrane complex providesthe necessary conditions for molecular connections, particularly laminin and collagen type IV that are essential for epithelialization. Studies have shown that matrix proteoglycans provide a source for growth factors, guide collagen aggregation and augment angiogenesis (27).

Electron microscopic and immunohistochemical studies have shown that by increasing SDS concentration, the decellularization rate of tissue increases but the amount of ECM compounds, such as carbohydrates decreases (26, 28).

In the current study, as with other studies, increased SDS concentration caused decreased GAGs content of the ECM and increased decellularization (Figs 4, 5). Related tissue engineering studies have also considered the density and porosity of the matrix. There is less cell migration in the matrix with a dense fibrillar collagen network in comparison to one with a porous fibrillar structure, because collagen acts as a physical barrier and inhibits cellular entry (29). The decrease in matrix porosity results in decreased food effluence and influence on matrix implanted cell growth (30).

By taking into consideration the increased porosity in the scaffold, the 1% SDS concentration had more advantages compared with the other studied concentrations. suggested that matrix elements influence cellular behavior. Thus, molecules such as collagen, fibrin, hyaluronic acid (HA) and laminin are used to prepare a suitable matrix. Additionally, collagen-glycosaminoglycan mixture and parts of the small intestine submucosal matrix are used as biomaterials (31).

In the current study the cells that migrated into the scaffold stained positive by PAS. These cells also stained positive with alcian blue and toluidine blue, but at a lessermagnitude (Table 2). These results probably indicatedthe presence of neutral carbohydrate compounds at higher intensity (in response to PAS) and acidic compounds (sulfated and carboxylated in response to alcian blue and toluidine blue) at lower intensity. The presence of blastema cells with such specifications in the scaffold showed that the prepared scaffold had suitable conditions for the migration and function of blastema cells. Thus blastema cells appeared to have the capability to be active and synthesize carbohydrate compounds in scaffolds *in vitro*.

The change in scaffold compounds due to the interaction with blastema cells was an interesting finding of this study, which was observed from week two and became more evident over subsequent days. Zones of blastema cells that migrated into the scaffold showed strongly positive response to PAS dye. Thus it could be concluded that the amount of neutral glycoconjugates in the scaffold probably increased due to the function of the cells. In addition, the average response to alcian blue and weak response to toluidine blue in various regions of the scaffold was probably an indicator of the discharge of acidic proteoglycans and GAGs into the scaffold. Spectrophotometry results with DMMB showed increased GAGs in the matrix (Fig 5).

In 2001, researchers cultured human gingival and dermal fibroblasts in collagen 3D scaffolds, with the intent to investigate matrix changes and study ECM discharge by these two types of cells. Their results revealed that matrix metalloproteinase (MMP) expression initially localized in the cytoplasm of cultured fibroblast cells before it became distributed in the ECM (32).

Veilleux and Spector (23) studied the effects of FGF2 growth factors on cultured chondrocytes in a collagen-GAG matrix in growth media. Spectrophotometry indicated that the GAGs content of the matrix in treated samples increased after two weeks.

In the current study we believe that at the time of blastema cell penetration these cells began to secrete compounds into the scaffold. According to histochemical and spectrophotometry analyses, these compounds included GAGs.

These observations further supported the prepared scaffold in this study that consisted of a 3D biological matrix which contained proper amounts of collagen and carbohydrate compounds. This particular scaffold might be employed for tissue engineering and grafts.

Collagen scaffolds can readily be degraded by the enzymatic activity of cytoplasmic lysosomes. Arg-Gly-Asp (RGD) peptide domains in collagen serve to maintain cell phenotype and activity. Matrix collagen increases cell adhesion and maturation (33).

Acellular dermal matrix (ADM), used in periodontology, is a cell- and vessel-free structure that needs receptor cells and blood vessels for reorganization (34). As cell-cell interactions and vessel structure are important in graft maturity, Rodrigues et al. (35) have cultured epithelial and fibroblast cells in ADM as one method for wound improvement. Seven days after culture, fibroblasts distributed on the ADM surface and created a transverse cell layer; however at 21 days after culture there was reduced cell density. Results showed that until 14 days, fibroblasts had proper conditions for cellular adherence and distribution within the matrix.

In this study blastema cells migrated into the matrix generated from human gingiva and extensively distributed in this scaffold. Different types of secreted glycoproteins were transferred into this matrix (scaffold) after synthesis by these cells and as a result, we observed aggregation of these compounds in both the cell membrane and ECM. Therefore histochemical results of this project are probably due to glycosylation function of proteins in migrated cells. The importance of carbohydrates in the scaffold possibly relates to their probable role as receptors of environmental and internal messages that direct cellular migration direction, amplification and differentiation in the process of restoration.

Polysaccarides, such as cellulose and GAG, most probably HA and proteins such as collagen, would be classified as natural scaffolds. The role of GAGs, which is composed of long carbohydrate chains, and their combination with collagen and perlecan in bone differentiation have been reported (36, 37).Cell surface carbohydrate chains and extracellular carbohydrate compounds are effective factors in cell actions such as migration, amplification, cellular identification, molecular targeting and cellular differentiation.

Carbohydrate manipulation in live cells and tissues is a fascinating new tool in tissue engineering and restorative medicine (38).

## Conclusion

The present study has shown that decellularized palatal gingival tissue (ECM) is a suitable scaffold for cell behavior studies. This scaffold may potentially be useful as a biological scaffold in tissue engineering applications.
